# Intima media thickness, pulse wave velocity, and flow mediated dilation

**DOI:** 10.1186/1476-7120-12-34

**Published:** 2014-08-23

**Authors:** Rosa Maria Bruno, Elisabetta Bianchini, Francesco Faita, Stefano Taddei, Lorenzo Ghiadoni

**Affiliations:** 1 Institute of Clinical Physiology, National Research Council, Pisa, Italy; 2 Department of Clinical & Experimental Medicine, University of Pisa, Via Roma, 67 560110 Pisa, Italy

**Keywords:** Carotid artery, Intima-media thickness, Arterial stiffness, Pulse wave velocity, Endothelium, Flow-mediated dilation

## Abstract

The identification of vascular alterations at the sub-clinical, asymptomatic stages are potentially useful for screening, prevention and improvement of cardiovascular risk stratification beyond classical risk factors.

Increased intima-media thickness of the common carotid artery is a well-known marker of early atherosclerosis, which significantly correlates with the development of cardiovascular diseases. More recently, other vascular parameters evaluating both structural and functional arterial proprieties of peripheral arteries have been introduced, for cardiovascular risk stratification and as surrogate endpoints in clinical trials. Increased arterial stiffness, which can be detected by applanation tonometry as carotid-femoral pulse wave velocity, has been shown to predict future cardiovascular events and to significantly improve risk stratification.

Finally, earlier vascular abnormalities such as endothelial dysfunction in the peripheral arteries, detected as reduced flow-mediated dilation of the brachial artery, are useful in the research setting and as surrogate endpoints in clinical trials and have also been suggested for their possible clinical use in the future.

This manuscript will briefly review clinical evidence supporting the use of these different vascular markers for cardiovascular risk stratification, focusing on the correct methodology, which is a crucial issue to address in order to promote their use in future for routine clinical practice.

## Introduction

Cardiovascular (CV) disease is the main cause of mortality and morbidity worldwide [1]. It has been suggested that risk estimation by classical risk factors (blood pressure, glucose, cholesterol) and circulating biomarkers may fail to adequately predict the risk of CV events because their fluctuations over time represent many transient and partial “snapshots” rather than a reliable picture of a complex situation developing over decades [2]. Conversely, imaging biomarkers of vascular damage might integrate the long-lasting cumulative effects of all traditional and non-identified CV risk factors and can be detected as target organ damage before clinical events occur, at a stage when interventions may be effective. For that reason, some of these biomarkers are recommended by international scientific societies for the improvement of CV risk stratification [3,4].

Recently, the concept of early vascular aging was introduced to describe structural and functional changes occurring in the large arteries with aging, which are accelerated in individuals at increased CV risk [5]. It can be measured by non-invasive techniques, and includes diffuse intimal thickening, usually measured as carotid intima-media thickness (C-IMT) [6], aortic stiffening, evaluated as carotid -femoral pulse wave velocity (cfPWV) [7], and endothelial dysfunction, commonly measured as brachial artery flow-mediated dilatation (FMD) [8,9].

Increased C-IMT is an intermediate stage in the continuum of atherosclerosis, which significantly correlates with coronary and cerebrovascular disease [10,11]. Other vascular parameters evaluating structural and functional arterial proprieties of peripheral arteries were then introduced. Increased cfPWV is already considered a subclinical target organ in hypertensive patients [4], since it has been shown to predict future cardiovascular events [12] and improve reclassification to a higher risk category [13]. Earlier vascular abnormalities, such as impaired FMD [8,9], have also been suggested for their potential use for risk prediction.

This review will focus on how to proceed to obtain accurate and reliable assessment of these vascular markers, improving their predictive value for the future use in clinical practice.

## Carotid intima-media thickness

C-IMT is measured by high resolution B-mode ultrasound of extra-cranial carotid arteries and it is the most widely accepted non-invasive marker of subclinical atherosclerosis [6,14]. C-IMT is considered an intermediate phenotype of atherosclerosis suitable for use in large-scale population studies [15], since its increase has been associated with higher cardiovascular risk [16,17] and with the presence of advanced stage of atherosclerosis in peripheral, cerebral and coronary arteries [18,19]. Epidemiological studies consistently reported a predictive value of increased C-IMT for myocardial infarction and stroke, independent of traditional CV factors [10,20-25], which has been confirmed in a meta-analysis of 12 relevant general population-based studies [26]. For these reasons, C-IMT is included in the in European Society of Hypertension guidelines as target organ damage (class II, level B) in hypertensive patients [4]. However, based on the results of a single study, the MESA [27], C-IMT is no longer recommended in the 2013 ACC/AHA Guideline on the assessment of Cardiovascular Risk [28].

C-IMT detected by high-resolution ultrasound represents the combined width of the intima and media, which are technically indistinguishable. C-IMT in healthy subjects consists almost entirely of media, with a progressive intimal thickening or medial hypertrophy determined by age, gender and hypertension, which do not necessarily reflect the atherosclerotic process [29].

The carotid artery is classified into three segments when undergoing ultrasound study, each approximately 1 cm in length. The most proximal segment, the common carotid (CCA) represent the 1-cm straight segment prior identified by a divergence of the near and far walls as the artery begins to divide into its internal and external branches. This focal widening of bifurcation extends over approximately 1 cm and is labeled the carotid bulb (CB). The tip of the flow divider separating the diverging internal carotid artery (ICA) and external carotid artery defines its distal margin; the final segment is the proximal 1 cm of the ICA. The CCA far wall is the easiest segment to be examined and the most commonly used measurement in clinical studies, resulting in a better prediction of stroke than myocardial infarction [11,30]. Several studies suggest the CCA far wall as the best location in terms of feasibility and reproducibility of C-IMT measure [31,32]. Nevertheless, it has been also suggested C-IMT measurements at multiple angles of both the near and far walls might provide the best balance between reproducibility, rate of C-IMT progression, treatment effect and their associated precision in this low-risk population with subclinical atherosclerosis [33].

**Figure 1:**
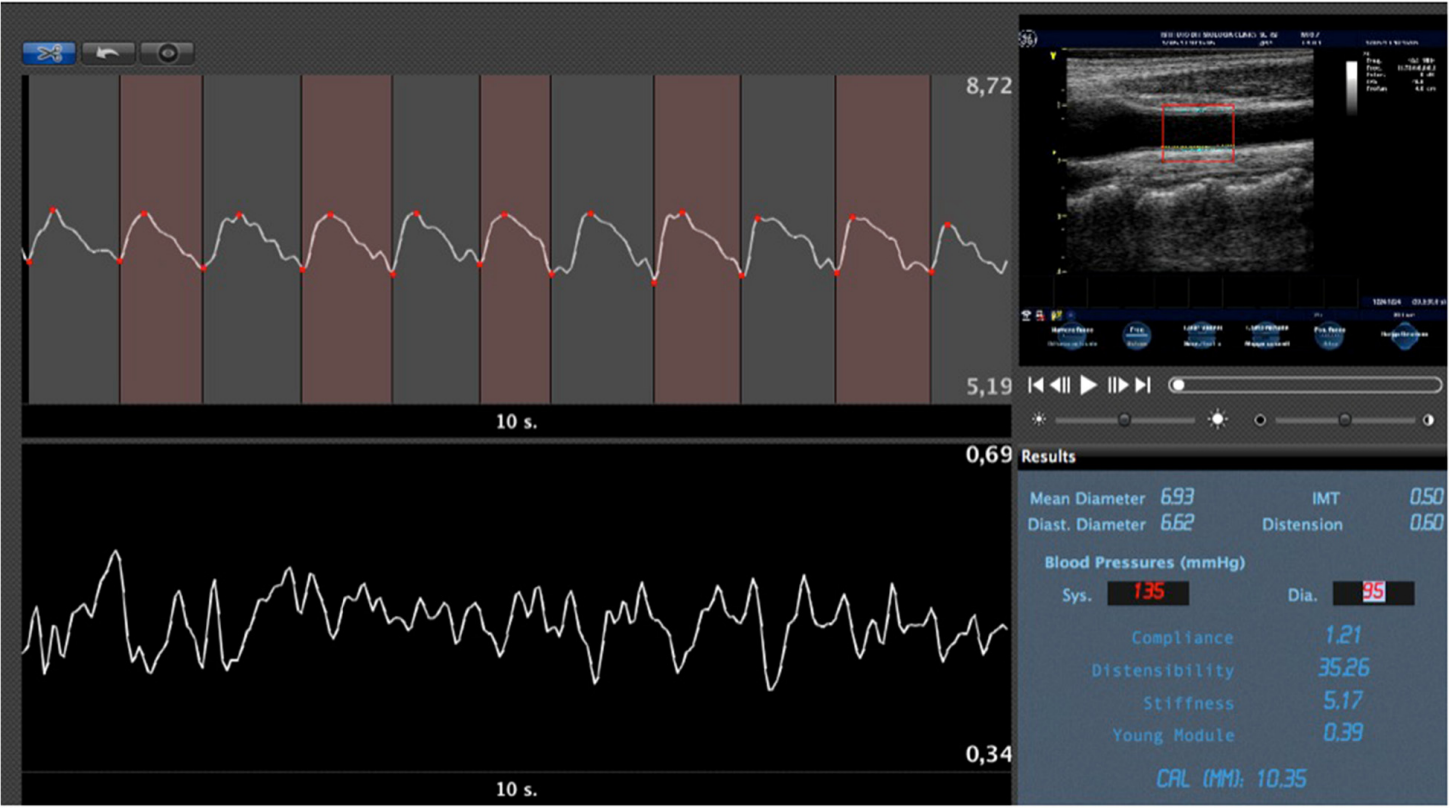
**Example of automatic edge detection of intima-media thickness (IMT) and diameter on B-mode scan of a common carotid artery (top right).** Graph shows changes over cardiac cycles of diameter (top) and IMT (bottom). The panel on bottom left reports mean values of diameter, IMT and some parameter of carotid stiffness.

Despite it has been proposed that a slower progression of C-IMT might be associated with a reduction in CV events [34], a meta-analysis including 41 trials with 18,307 participants showed no significant relationship between C-IMT regression and events, suggesting that regression or slowed progression of C-IMT, induced by CV drug therapies, may not reflect a reduction in CV events [35]. However, it has to be acknowledged that the estimation of C-IMT in the vast majority of the studies was obtained manually by calipers, without the use of an automatic approach for the measurement. Therefore, accuracy of C-IMT and, particularly, of its changes over time is not simply a methodological question but might have important consequences in the clinical setting. B-mode image processing-based devices (Figure 1) and radio-frequency (RF)-based echo-tracking systems are now widely available and should definitely be adopted to increase precision and accuracy. RF-based devices are considered very accurate since they are based on signals with higher spatial resolution than B-mode data [36,37]. However, robust image-based systems have been recently validated, showing similar reproducibility [38,39]. These systems require high quality of the scans and standardized system settings (e.g. dynamic range, depth gain set or filtering) [40], that should clearly reported, especially for follow-up studies [41]. The reliability of B-mode based systems has been recently acknowledged by the scientific community, since measures obtained by this technique were used for the definition of C-IMT reference values obtained by echo-tracking [42]. C-IMT reference values were obtained by echo-tracking in a cohort of 24871 individuals from 24 research centers worldwide [42], and allowed estimation of C-IMT age- and sex-specific percentiles in a healthy population of the 4234 individuals without CV disease, CV risk factors, and blood pressure-, lipid-, and/or glucose-lowering medication. These reference values will favor the use of C-IMT assessment in clinical practice, possibly for a better risk classification particularly concerning C-IMT modifications over time.

## Pulse wave velocity

Arterial distensibility is a measure of the artery’s ability to expand and contract with cardiac pulsation and relaxation. Aging, hypertension and other risk factors can alter the structural and functional properties of the arterial wall, leading to a decrease in arterial distensibility, which seems to be a common pathologic mechanism among CV diseases [37]. The aorta is a major vessel of interest when determining arterial stiffness, since thoracic and abdominal aorta makes the largest contribution to the arterial buffering function [37]. Elastic properties of conduit arteries vary along the arterial tree, with proximal arteries being more elastic and distal ones stiffer. Aging, along with blood pressure, is the main determinant of stiffness in both carotid and aortic stiffness [43,44], inducing a reduced synthesis and an increased degradation of elastin, and on the other hand to an increased synthesis and reduced degradation of type 1 and type 3 collagen [45].

Aortic degeneration is responsible for most of the pathophysiologic effects of central pressure and arterial stiffness on the left ventricle, brain, and kidney [5,7,37]. The assessment of PWV is considered to be the “gold standard” measurement of aortic stiffness, as it is a simple, non-invasive and reproducible method and has the largest amount of clinical evidence, providing the predictive value of aortic stiffness for CV events [12].

**Figure 2:**
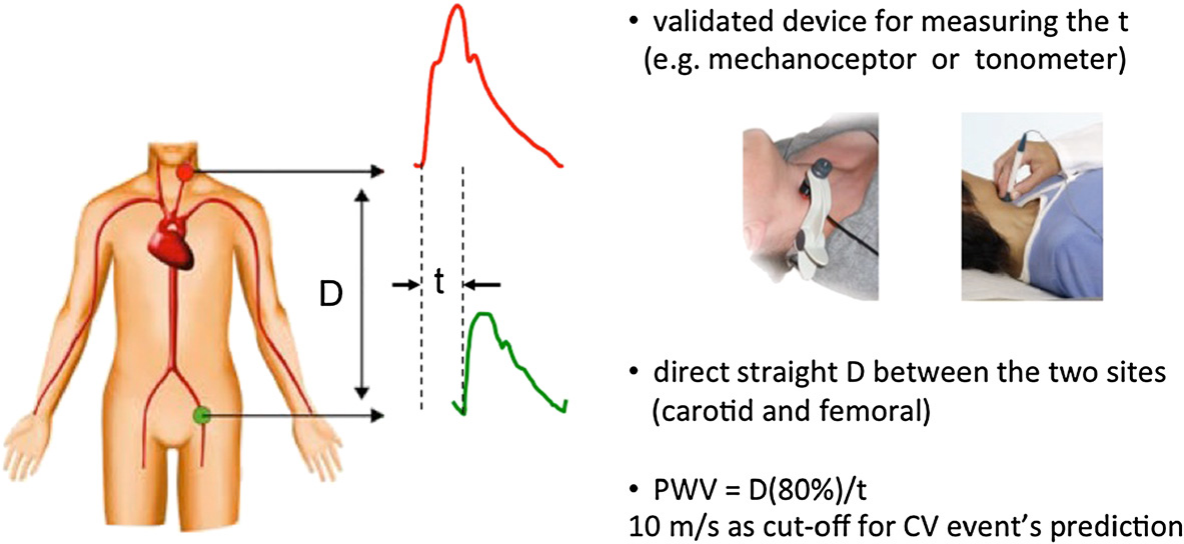
**Figure shows a schematic representation of pulse wave velocity (PWV) according to current international recommendations **[48]**.** Pulse wave travel time: t; distance: D; CV: cardiovascular.

PWV can be measured from various arterial sites: the pressure waveforms are usually obtained trans-cutaneously at the common carotid artery and the femoral artery. The distance covered by the waves is assimilated to the surface distance between the two recording sites, and the time delay (or transit time) measured between the feet of the two waveforms is estimated. PWV is then calculated as the ratio of the distance to the time delay: *PWV = (distance/transit time)* (Figure 2).

Distance has to be measured precisely, since small inaccuracies may influence the absolute value of PWV [46]. Inaccurate distance measurements might occur with abdominal obesity, particularly in men [47]. In the past decade, different modalities for aortic path estimation have been used, leading to heterogeneous and non-comparable results among different research groups, but very recently, accumulation of data using magnetic resonance imaging allow the definition of a shared methodology in all the laboratories [48].Transit time is commonly estimated by the foot-to-foot method (Figure 2). The foot of the wave is defined at the end of diastole, when the steep rise of the wave front begins. The transit time is the time of travel of the foot of the wave over a known distance.

The most accepted methods for cfPWV measurement are those using mechano-transducers or applanation tonometers (Figure 2). Pressure waveforms can be recorded simultaneously to provide automated measurement of PWV as with the Complior System (Colson, Les Lilas, France) which employs dedicated mechano-transducers directly applied on the skin [49]. The transit time is determined by means of a correlation algorithm between each simultaneous recorded wave. Pressure waves can also be recorded sequentially from different sites by a single high-fidelity applanation tonometer, and transit time calculated using registration with a simultaneously recorded ECG, such as with the SphygmoCor system (AtCor, Sydney, Australia) [50]. Brachial-ankle pulse-wave velocity (baPWV) has been suggested as a simpler alternative to cf-PWV, since the aortic PWV was the main independent correlate of baPWV, followed by leg PWV [51]. Finally, the distension waves obtained at a short time interval at two arterial sites (common carotid and femoral artery, for instance) by high-resolution echo-tracking devices can be also used to calculate PWV, using the R-wave of the ECG for calculating the time delay [37].

In 2010 the Reference Value for Arterial Stiffness Collaboration provided normal and reference values for cfPWV, calculated using a standardized methodology in a large European population (data from 16 867 individuals and patients from 13 different centers across 8 European countries) [52]. Values were defined in a ‘normal’ population of individuals having optimal blood pressure values and no additional CV risk factors, whereas ‘reference’ values were identified in individuals or patients presenting CV risk factors. For normal and reference values, the population was categorized according to age decade and BP category.

As already mentioned, current hypertension guidelines include cfPWV in the list of factors influencing the prognosis of hypertensive patients: a threshold cfPWV value of greater than 10 m/s is considered as an index of large artery stiffening and an indicator of sub-clinical organ damage [4]. This value derives from a standardized calculation for PWV, which takes into consideration the most accurate approximation of the distance between the carotid and femoral artery [48]. However, it is still discussed whether this threshold or a series of threshold values (such as **>** 90th percentile or **>** 75th percentile of normal values for PWV dependent upon age) would be more accurate reference values for identifying people at increased CV risk [53].

## Flow-mediated dilation

Endothelium plays a primary role in the control of vascular function and structure, by production and release of nitric oxide (NO) under the influence of agonists and by mechanical forces, such as shear stress [54]. NO not only cause vasodilation, but also inhibits key mechanisms of vascular damage, such as platelet aggregation, vascular smooth muscle cell proliferation and migration and monocyte adhesion [54,55]. Despite its key importance in vascular pathophysiology, NO can be hardly assayed directly, for its biochemical properties. For this reason, several indirect methods for assessing stimulated NO release, and thus endothelial function, have been developed [9,56,57].

Endothelial function in clinical research is usually tested by vascular reactivity studies [56], measuring the degree of vasodilation as the changes in diameter induced by specific stimuli in the macrocirculation (e.g. in the epicardial or brachial arteries) and the microcirculation (coronary, peripheral muscle, subcutaneous and skin microcirculation).

**Figure 3:**
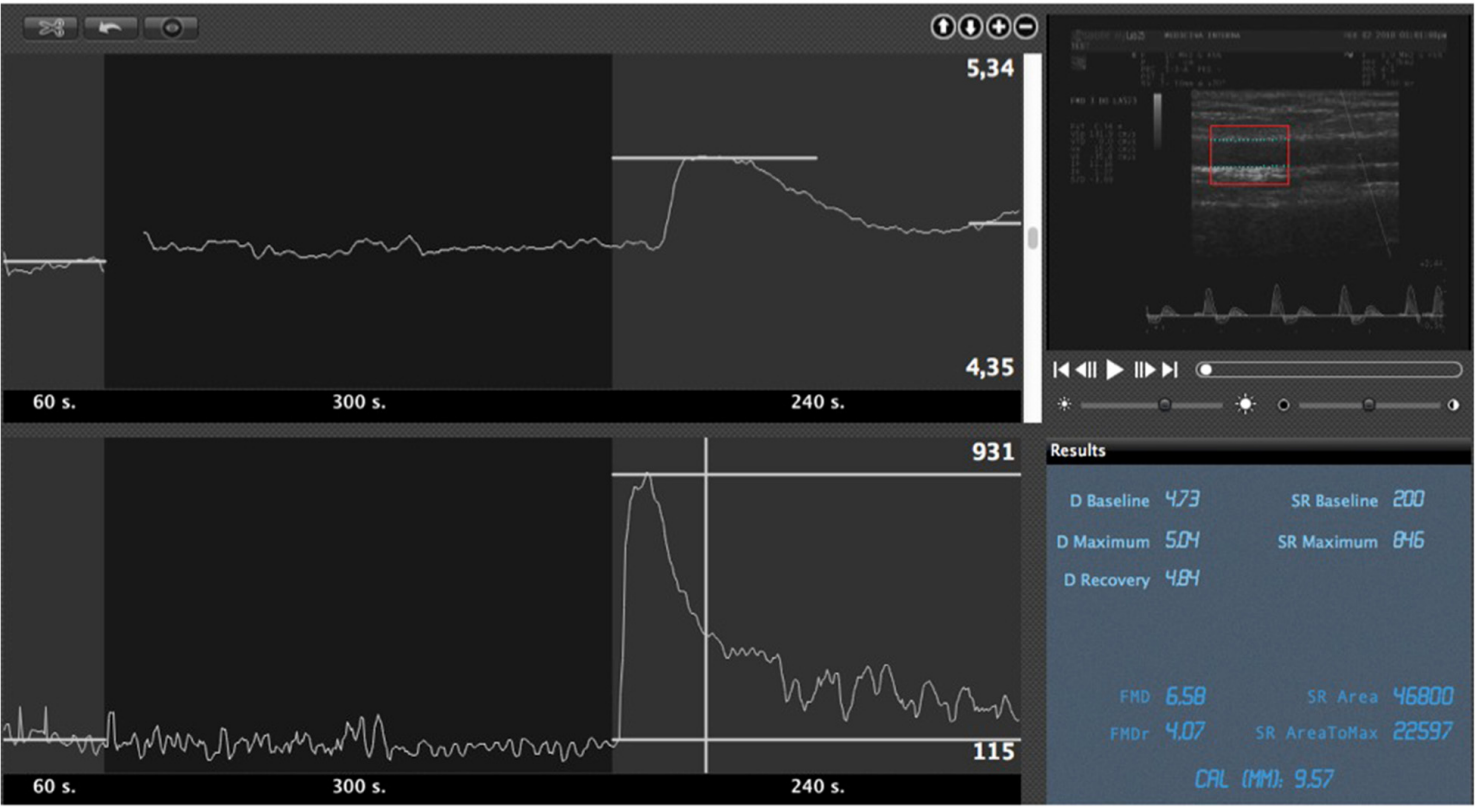
**Example of automatic edge detection and real-time analysis of B-mode and doppler signal during flow mediated dilation (FMD) assessment (top right).** Graphs show changes in brachial artery diameter diameter (D, top panel) and shear rate (SR, left). The panel on bottom left reports mean baseline, maximun) values of D and SR, and the computation of FMD.

FMD is a non-invasive method, introduced in 1992 [58], which is based on the measurement of brachial artery diameter changes after an increase in shear stress induced by reactive hyperemia (Figure 3). The NO-dependency of FMD has been documented invasively with intra-arterial infusion of by N^G^-mono-methyl-L-arginine (L-NMMA), a specific inhibitor of NO synthase, which reduced of almost 2/3 the dilation of the artery (radial or brachial) in response to shear stress [59,60].

Available data indicate that impaired FMD is associated with almost every condition predisposing to atherosclerosis and CV disease, as a putative intermediate step for the development of subclinical target organ damage and later clinical events. Cross-sectional studies have shown a correlation between FMD and C-IMT [61,62] and C- IMT progression over a 6-year follow- up in a population free of cardiovascular disease [63] or after 1 year, in hypertensive, postmenopausal women [64]. Impaired FMD has been shown to be an independent predictor of in-stent stenosis after single-vessel coronary interventions [65]. In the setting of primary prevention, most of the studies showed that FMD is predictive of cardiovascular events beyond traditional risk factors in special subsets of patients such as after elective vascular surgery [66], in patients with chest pain [67], in hypertensive patients [68,69] and in large population studies [70,71]. A recent meta-analysis summarized the results of four population-based cohort studies, and ten cohort studies, involving 5,547 participants, showing a pooled relative risk of cardiovascular events per 1 % increase in brachial FMD, adjusted for confounding risk factors of 0.87 (95% CI, 0.83-0.91), which was consistent among all subgroups evaluated [72]. Robust evidence on the effect of non- and pharmacological interventions on endothelial function is available, in particular with antihypertensive treatments [73]. However, only two studies suggest that postmenopausal hypertensive women [74] and patients with stable coronary artery disease [75] not responding to the interventions with an FMD improvement are at considerable higher risk of further events. In a direct comparison of different biomarkers in a cohort of 1330 intermediate-risk participants of the MESA study, FMD failed to demonstrate increased discrimination and risk classification in comparison to a standard approach [27]. However, this negative result does not put an end to the debate, since FMD reproducibility in the MESA study was quite low [27] in comparison to state-of-the art approaches [76,77], stressing even more the importance of a correct methodology when approaching this technique.

FMD implies the measurement of brachial artery diameter before inflation (to 200–300 mmHg) and after release (5 minutes later) of a sphygmomanometer cuff placed on the forearm [78]. Despite the apparent simplicity, its application is technically challenging [8,56,57]. Indeed, some important caveats should be considered, for the effect of environmental or physiological influences [79] and technical variability of its measurement, which requires standardization [8,78,80].

Variations in the technique, such as the position of the occluding cuff at the arm instead of the forearm may produce heterogeneous results that are less representative of local NO activity [8,57], although possibly with the same prognostic significance [81]

The application of a rigorous methodology is able to reduce FMD variability in single center [82] and multicenter studies [76,77]. This comprises certified operator training [78] and defined experimental settings, including adjustable stereotactic probe-holding device and automated computer-assisted brachial artery measurements [83,84], that should be implemented in all FMD laboratories. In particular, automated real-time systems for diameter measurement are able to enhance reliability of FMD (Figure 3), by reducing the number of rejected examinations due to poor quality and/or instability of the images [76,85] and improving operator’s learning curve during training and certification [86].

Although the FMD methodology was set up more than 20 years ago, it is still evolving in terms of study protocol, variables considered and mathematical analysis. Alternative computation of FMD, independent of diameter, has been recently suggested [87], since the larger is the diameter, the smaller are the relative percent changes [80]. The issue of the assessment of endothelial function not only in the hyperemic state has been also introduced by measuring vasoconstriction during cuff inflation (low-flow–mediated constriction, FMC) particularly at the level of the radial artery [88]. Another important aspect that is gaining growing attention is the acquisition of stimulus for FMD itself, such as the reactive hyperemic flow, and the induced shear stress, which might be an important measure of peripheral microvascular function [80,89,90]. Finally, the evaluation of endothelium-independent dilator response should be always tested by the administration of low-dose sublingual nitroglycerine [91], to exclude the possibility that altered smooth muscle cell contractility may influence the endothelium-dependent response. Furthermore, the evaluation of smooth muscle cell function as part of the FMD protocol might also convey additional information about vascular health in patients with CV risk factors and disease [92].

## Conclusions

Prevention of CV disease requires risk stratification and treatment of classical risk factors, such as smoking, hypertension hypercholesterolemia and diabetes. Nevertheless, classic risk stratification might be not sufficient to provide an accurate estimate of probability of future CV events.

Vascular biomarkers, which are parameters of subclinical cardiovascular disease, could increase the estimation of the individual cardiovascular risk and improve strategies for effective prevention.

Several vascular markers obtained by ultrasound and other non-invasive techniques have been proposed for this aim. Greater evidence is available for increased C-IMT and cf-PWV, which are able to improve risk reclassification and can be used to identify subclinical target organ damage. FMD only recently has reached an adequate level of methodological standardization to be proposed as surrogate endpoint in clinical trials. A solid methodology will allow to verify the intriguing hypothesis that assessment of changes in vascular biomarkers after therapy might relate to the development subclinical target organ damage or events in future clinical trials.

## Competing interests

Elisabetta Bianchini, Francesco Faita and Lorenzo Ghuadoni are co-founders councilors of QUIPU s.r.l., Pisa, ITALY.

## Authors' contribution

RMB and EB drafted the manuscript. FF, ST and LG reviewed the manuscript.

All authors read and approved the final manuscript.
